# The Beginning of a New Era: Artificial Intelligence in Healthcare

**DOI:** 10.34172/apb.2021.049

**Published:** 2020-07-15

**Authors:** Akshara Kumar, Shivaprasad Gadag, Usha Yogendra Nayak

**Affiliations:** Department of Pharmaceutics, Manipal College of Pharmaceutical Sciences, Manipal Academy of Higher Education, Manipal, Karnataka 576 104, India.

**Keywords:** Artificial intelligence, Healthcare, Drug discovery, Surgical robotics, Deep learning

## Abstract

The healthcare sector is considered to be one of the largest and fast-growing industries in the world. Innovations and novel approaches have always remained the prime aims in order to bring massive development. Before the emergence of technology, the healthcare sector was dependent on manpower, which was time-consuming and less accurate with lack of efficiency. With the recent advancements in machine learning, the condition has been steadily revolutionizing. Artificial intelligence (AI) lies in the computer science department, which stresses on the intelligent machines’ creation, that work and react just like human beings. Currently, the applications of AI have been expanding into those fields, which was once thought to be the only domain of human expertise such as healthcare sector. In this review, we have shed light on the present usage of AI in the healthcare sector, such as its working, and the way this system is being implemented in different domains, such as drug discovery, diagnosis of diseases, clinical trials, remote patient monitoring, and nanotechnology. We have also briefly touched upon its applications in other sectors as well. The public opinions have also been analyzed and discussed along with the future prospects. We have discussed the merits, and the other side of AI, i.e. the disadvantages in the last part of the manuscript.

## Introduction

**Figure 1:**
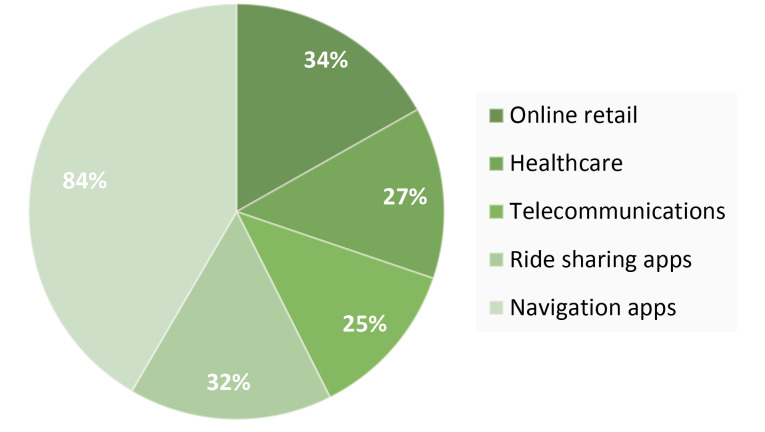
Use of AI is used in different domains.

According to the recent status, it has been observed that the AI tools are being used in the major areas of early detection, diagnosis, treatment, the prediction of the outcome for diseases like cancer, neurology, cardiology, pathology etc.^1^ AI has been designed in a manner to provide assistance to the physicians, to help them take better clinical conclusions or in some cases, replacement of the judgements made by them in some of the functional areas pertaining to healthcare. It uses intricate algorithms and has the capability to self-correct for enhanced accuracy. The system uses advanced techniques in order to predict health outcomes and health risk alerts by the extraction of vital information from a large population of patients. AI has progressed with the development of deep neural networks, natural language processing, computer vision and robotics.^2^ The physicians find it extremely useful, as they are provided with the full information from various sources of journals, textbooks, medical magazines etc. This helps them in the reduction of diagnostic and therapeutic errors. Though the coining of the term- ‘Artificial Intelligence’ dates back to the year 1956,^3^ the popularity and the usage emerged in recent times, due to the advancement in the algorithms, improved storage capacity and the power of computing. Initially, it was involved in basic solving of problems and the symbolic methods. Of late, it has advanced to imitate the humans basic reasoning. In the recent times, the common usage of processed personal assistants like Siri (iOS), Google Assistant (Android), Cortana, Alexa is spreading in almost every household. The common man visualizes AI as some scary human-like robots taking over the world as depicted by the Hollywood movies and the fictional novels. But, in reality it has evolved to provide benefits to the industries. AI has been implemented in almost every facet. The fascinating fact is that it doesn’t function on a constant pre-installed program and algorithms, but adapts itself to the new progressed algorithms learning to prevent it from becoming outdated. AI is not a separate application or a commodity that can be sold, rather it is an addition of its intelligence to the existing objects, like the products that we already use, improvised with AI capabilities. For example, Siri was a newly added feature to the Apple products. Similarly, Google Assistant for the Android products. AI has been paving its way into the healthcare sector, mainly in diagnosing certain life-threatening diseases like cancer, neuronal and cardiac diseases, wherein early diagnosis is a very crucial factor.^4^ Apart from these diseases, it is being used for the diagnosis of congenital cataract disease and diabetic retinopathy.^5^ The implementation of AI in manufacturing industries helps to provide safer operational environment, which can further enhance the quality and the quantity of the production.^6^ The pioneering AI was deployed by IBM Watson, who brought in a major fundamental change to healthcare industry.^7^ The extent to which AI has been used in different domains has been depicted in Figure 1.^8^

## How does AI work?

**Figure 2:**
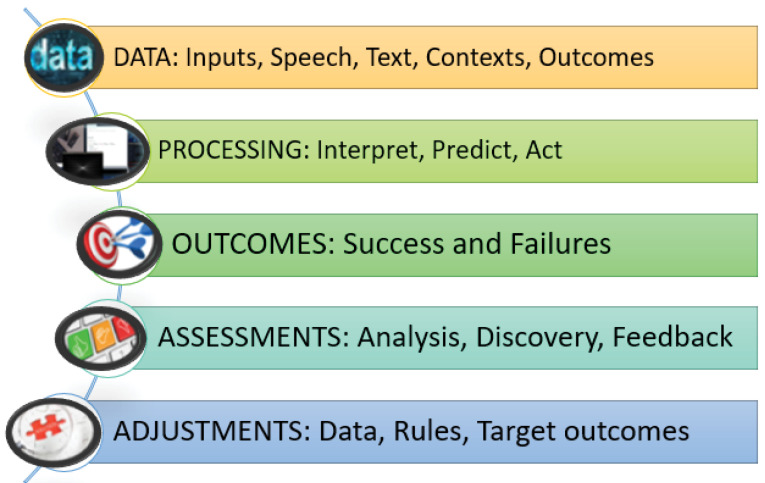
Working of AI process.

AI functions with a combination of vast amounts of data with quick and intelligent algorithms, which allows the software to learn spontaneously from the patterns or from the data features. Implementation of AI into the machines is basically program based. A designed program is installed, which contains the information as to how the function has to be carried out. It works on the combination of huge amounts of data with quick, repeated processing and intelligent algorithms.^9^ Generally, AI analyses the surroundings and accordingly acts with the pre-installed algorithms and program. This increases the chance of success. All the information is stored in the ‘Cloud’. ‘Cloud’ is a storage platform, which has the capability to store tons of data, statistics and information which can be accessed via internet. This helps the system to easily function with high speed and accuracy.^10^ The working of AI has been depicted in Figure 2.^11^ AI plays a key role in developing not only business and its processes, but also the humans to a different level. With such a rapid growth in technology and its progress, one can expect even more mesmerizing features and uses of AI in the future.

## Objectives of AI

One of the main goals of AI is problem solving. It works by collecting the information through the inputs provided by human and sensors. Then, it compares the provided information that is already stored in the computer for the determination of the significance of the provided input. According to an article published by the Computer Science Department of UMass Amherst, computers can solve the problems that is programmed for it to solve or if it has the required information for the same.^12^ Apart from problem solving, another goal of AI is to learn. Some of the intelligent systems are able to bring in the preferred outcomes or able to conquer a hurdle in an uncommon situation by undertaking different paths and then remembering that path that was undertaken for that particular situation that had been successful. So, when it come across a similar situation in the future, it can handle it in the best possible manner.^13^ A fine example for this, is the usage of the feedback system. In terms of any difficulty for the user, and the system could not respond at that particular moment, it will take a feedback, work on it and then remember it for the future. AI majorly aims to render the tasks automated so that it can reduce the time and efforts involved otherwise.^14^ It aims to analyze and then decipher the working mechanisms in the brain and then translate this knowledge to AI architectures (which can be implementable) with the goal to evolve in a more efficient and flexible technical system. Other main aims include:

(a) Creation of expert systems, which include the system having the ability to learn, think, show intelligent behavior and supply the users with the best possible advices.

(b) Execute intelligence in machines that can understand, learn and behave just like humans.^15^

**Figure 3:**
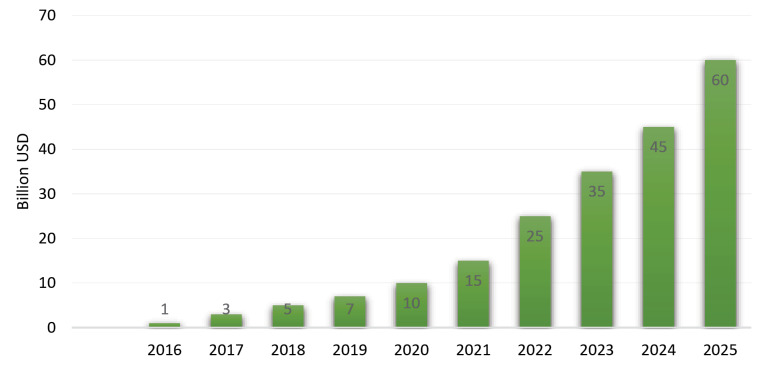
Estimated revenue generation from AI over the years.

AI is one of the most progressing systems in all the domains. Almost all the fields have some or the other applications of it. The sectors like the financial, service, banking, computer games, fraud detection, virtual assistants in the form of chatbots etc, are utilizing AI.^16^ However, the implementation of AI in healthcare industry is still at its infancy. Not all the facets in healthcare has fully implemented the usage of AI. According to the scientists, a few years down the line, AI would be the most adaptable and dependable technique on this planet, and user-friendly.^17^ In the year 2011, IBM’s estimation about the whole of the healthcare domain was a whopping 161 billion GB of data!^18^ With such an immense amount of data availability, AI has been recorded to become a game-changer for the improvement of care and also to curb the current trend of unsustainable healthcare. Due to the fact that AI is still developing, the aims keep changing and updating. AI is turning into a hot prospect in the field of technology. According to Statistica, it has been estimated to obtain a revenue of about $60 billion by the year 2025^19^ (Figure 3).

## Applications of AI in healthcare

### Drug discovery

One of the most important baseline for a pharmaceutical industry is the drug discovery. The average time taken for a drug to reach the market is about 14 years, with a whopping cost of an average $2.6 billion.^20^ Moreover, the selection of a new successful drug molecule is the toughest task from the cluster of prospective pharmacological active chemical entities (lead molecules). With the help of AI, researchers are able to select few clinically effective candidates out of thousands of molecules with the help of computer- aided drug design software in a lesser amount of time,^21^ whereas, traditional methods may require years together. AI involves the networks which recognize some strategies in drug molecule design and accordingly algorithms are designed, which aids in understanding the differences between normal and disease profiles within a complex set of data.

**Figure 4:**
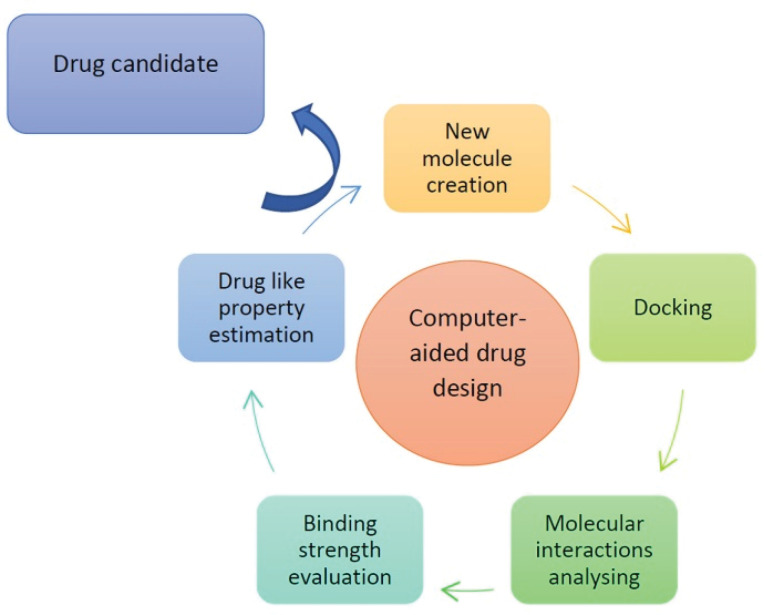
Process of computer- aided drug design.

Implementing AI has reduced the time by many folds and the development can be done within 2-4 years.^22^ The process of computer- aided drug design has been illustrated in Figure 4.^22^

Recently, in the year 2015, there was an epidemic outbreak of Ebola virus (West African), which struck many countries, killing 11 310 people^23^ (in the United States, some African and European countries). It almost had the potential to turn into a global pandemic. At this point, there was an urgent need for a drug which could be used for its treatment. So, an AI-powered program was used to screen the drugs available which can be re-designed to combat this disease. A company called- Atomwise collaborated with the Toronto University and IBM to identify a treatment for Ebola virus infections. To perform the drug research, core AI technology was required, which was provided by Atomwise. The Toronto University researchers provided the information of the biological details of the virus, while IBM provided their Supercomputer- 64 000 Blue Gene/Q (Blue gene is an IBM project which aims to design supercomputers that reaches high operating speeds with minimal power consumption). Then, a screening was conducted for the molecules which binds to the glycoproteins. The compounds already had an inbuilt data for safety for use in the patients and was proposed to be quickly brought into clinical trials use. Using the system, they were able to analyze and ascertain that binding affinity of the molecules to glycoprotein. The Toronto University then tested the compound *in vivo*and identified that the compound was found to have no previous antiviral activity. These studies revealed that, the Ebola virus’ glycoprotein 2 is important for its viral entry and its fusion, and this protein had 3 outer helices. It was recommended to block the conformational shift of this glycoprotein, which could potentially block the viral entry. Thus, the drugs were made in a short span of time, and a global pandemic could be prevented.^24^

After the discovery of a drug, it generally takes 4.5-5 years for it to reach the clinical trials stage.^25^ Astonishingly, in 2019 a Japan based pharmaceutical company, Sumitomo Dainippon Pharma which had a collaboration with UK based biotech- Exscientia designed a new drug using AI for the treatment of OCD (obsessive-compulsive disorder). It is the first in the medical world to design a drug using AI ri ght from the scratch; the task which takes 4.5 years was achieved within 12 months.^26^ The AI adopted by Exscientia uses a range of algorithms to determine a new lead molecule. The molecule was named as DSP-1181 which is a long acting 5-HTA1 receptor agonist.^27^ The main aim was to target a specific receptor present in the brain, which is involved in OCD.^27^ Against a huge range of diseases such as oncology, cardiovascular diseases) and other rare diseases, the algorithms could be applied to any of the targets where the drug could act. Exscientia is currently working with the other pharmaceutical companies like Bayer and Sanofi for designing drugs for other metabolic diseases.^28^

Another example of AI usage is the recognition of cardiotoxic and non- cardiotoxic drugs belonging to the anticancer category by the usage of an AI algorithm.^29^ Machine learning has also been explored for the identification of potential antibiotics from a list of 100 million molecules. Halicin is the first antibiotic to be identified using AI.^30,31^ The use of these algorithms could also be used to identify molecules which could combat antimicrobial resistance. As antibiotic resistance is on the rise and is a threat to millions of people, scientists are working on ways to overcome this challenge. AI has been explored by scientists in studying the DNA sequences which are responsible for causing antibiotic resistance.^32^

### AI in diagnosis

Upto 80 k deaths are reported every year due to the misdiagnosis of illness.^33^ Large number of cases and incomplete medical histories has led to deadly errors. AI is immune to these variables. When compared to medical professionals, AI has an ability to predict and diagnose illness at a faster rate.^34^

AI has been widely explored in the diagnosis of cancer. The early diagnosis as well as the prognosis is of utmost importance in improving the survival rate in cancer patients. Advances in the field of computers has led to the use of AI in analyzing the prognosis of the disease. Ichimasa and group studied the use of AI in ascertaining the need for surgery after the resection of T1 colorectal cancer. In practice, the prediction of lymph node metastasis is challenging prior to surgery. Hence, the lymph node metastasis was analyzed using AI model based on pathological results. The results were compared to that in the guidelines which suggested that AI may be successfully adopted in avoiding unnecessary surgeries.^35^ Coudray et al utilized AI (deep convolution neural network) to study the mutations from the histopathological results of non-small cell lung carcinoma. Their study concluded that the hospital visits of patients could be reduced by the automatic classification of nodules.^36^ Studies using deep learning techniques revealed that the various non-Hodgkin lymphomas could be differentiated based on the results of cluster and discriminant analyses.^37^ Deep learning algorithms have also been successfully utilized in detecting the lymph node metastases in breast cancer patients based on the pathology results. The data obtained was validated based on the results obtained from pathologists.^38^ The early detection and predictive assay in the precision oncology have helped in the stratification of patients for the treatment. This has become possible with the help of digital pathology which is an AI based computational approach. The companies like PathAI, Proscia, PAIGE.AI, Inspirata and DeepLens are using AI based tools for diagnosis, prognosis detection of different cancer subtypes.^39^

### AI in surgical robotics

Surgical robots have gained wide applicability in more than 1 million surgical procedures pertaining to orthopedics, gynecology, neurology, oncology, dentistry. AI has been amalgamated with surgical robotics which has enabled exchange of important information between the surgical robots. AI could aid the surgeons in gaining access to real-time warnings and provide advice during the procedure. Deep learning data could be utilized to render the best surgical practice with accuracy.^40,41^ However, this clinical AI requires further validation to achieve the best outcomes. AI have been used in conjunction with robots to aid in the precise withdrawal of blood from minute blood vessels during the clinical procedures.^42^

### Boost in clinical trials

In this technology driven age, companies never leave an opportunity to increase the efficiency and betterment in their business processes. Pharmaceutical companies also are not exception in this case. Sensing the promising future ahead, the companies are largely investing in AI as the application has made their processes efficient, accurate and seamless. In the early 2000s, i.e., from 2000 to 2015, the success rates for clinical trials were quite low. It was reported that only 13.8% of the candidates could successfully get through all the three stages of clinical trials.^43^ After the implementation of AI, it has reduced the cycle time and at the same time, improved the productivity costs as well as the outcomes. The AI algorithms along with a digital infrastructure, enables a continuous stream of clinical trial data to be coded, stored and managed.^44^ Apart from this, electronic data capture is being used in an improved manner for the reduction of human errors for the collection of data and integrate with other databases seamlessly. The technologies enabled with AI collects, organizes and analyses the increasing amount of data which gets generated by clinical trials, which includes the failed trials and helps in the extraction of meaningful patterns of information that aids with design. Details like EMR (electronic medical records), medical images, which were collected through the physicians’ notes are utilized to determine the appropriate patients for the trial procedure.^45^ Additionally, it has also become easier for the patients who can convey the changes and other information through their smartphones and wearable devices without much effort. After monitoring the patients continuously and sharing the data across systems, all the data is consolidated on an analytics platform. A self- learning system which has been designed to provide the real-time insights about the safety and efficacy of the treatment and predicting the dropouts risk, such that the engagement and retention can be enhanced.^46^

**Figure 5:**
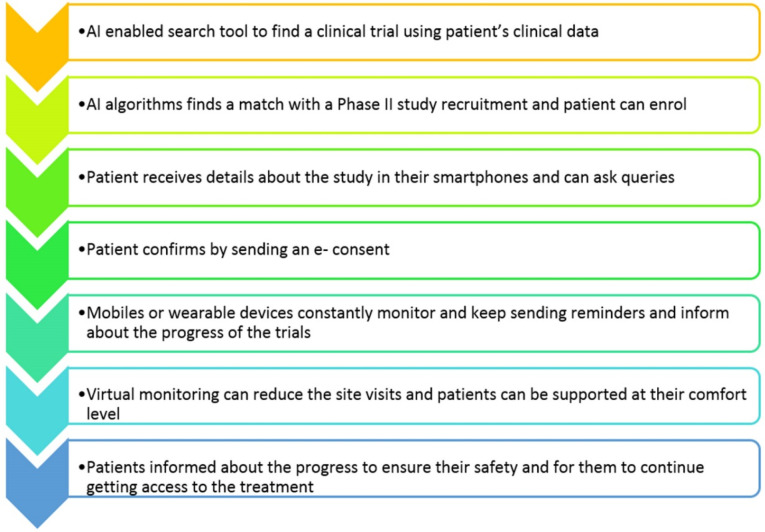
Journey of patients in an AI- enabled clinical trial.

In the coming future, the biopharma companies have planned for the development of tailored therapies that can treat the diseases rather than their symptoms. The usage of AI- enabled health technologies and the patient support platforms have been predicted to revolutionize the clinical trials with improved success in the attraction, engagement and retaining of committed patients throughout the study duration. A step- by- step process of the digitalized clinical trial has been explained in the form of flow chart (Figure 5).^47^

Companies like AiCure and Brite Health have implemented AI in order to monitor the patients on the way they react to the treatment during clinical trials, with the usage of audio and visual data to ascertain the efficiency of the treatment and for increasing the adherence to the trial procedures and retain the patients by preventing dropouts.^48^ Unlearn.ai, a San Francisco based start-up has been trying to reduce the number of candidates for the trials by using digital twins, which is a replica that can be used for various purposes.^49^ This leads to a reduction of the possibility of resistance in patients, by reducing the risk of the patients getting assigned to placebos as well as decrease the number of candidates required for the completion of the trial.^50^ Vendors likeTrials.ai, Antidote.me and Deep 6 AIhave developed AI applications that increases the recruitment process by making it comfortable for the patients to enroll and engage and also helps in the analysis of the medical records for the identification of patients who may be suitable for the trial.^51^

### Digital consultation

The concept of digital consultation is to reduce the hospital visits of the patients for minor symptoms, which can be self-treated with the assistance of a medical practitioner within the comfort of their homes. The apps use AI to provide the consultation, which is based on the patient’s medical history(which can be found with a questionnaire that the patient would be requested to fill) and the general knowledge related to the medical field.^52^ The users are required to feed in their symptoms in the app. The speech recognition compares the symptoms provided by the with the databases of the illness. Recording the patient’s medical history, the system recommends the course of action. One of the popular apps which was founded in 2013, Babylon, a UK based app extensively used for digital consultation purpose.^53^

A study was conducted which revealed that, almost around 45% of the population falling in the age groups of 18-29 years did not have a doctor whom they visit regularly for their illness.^54^ Apart from this group, people who have tight work schedules, find it quite difficult to squeeze in time to visit a doctor often resort to self- treatment or sometimes go for over-the-counter medicines.^55^ Keeping in mind the popularity of the digitalized world, apps like Buoy chatbots were introduced in the year 2015, using integrated AI system. It has a symptom checking chat-box, which uses interactive methods with the patients and is a well-organized digital consultation tool. Buoy uses pre-determined responses to the patient. Based on their health concerns, the users can choose from the options given in the app. Buoy has the intelligence (powered by AI) to determine if the patient’s health issue is of major concern, which will then prompt the patient to seek medical help and also aid the patients in finding the right physician near their locality, thereby reducing the time and efforts of the patients to find the clinics out of their way.^56^

### Remote patient monitoring

It is a known fact that, the main source of expenditure in a hospital is the manpower. To reduce this cost and at the same time providing the patients with the required services, the concept of remote patient monitoring came into existence. As AI, sensors and the predictive analysis are getting more advanced, there has been rapid developments in the concept of patient monitoring. Wearables and embedded sensors like the glucometers, blood pressure monitors etc are already available. Apart from all this, there are much more advances like smart implants, smart prosthetics which are used post -surgery or for rehabilitation in patient management.^57^ It helps in avoiding post-operational complications by continuously monitoring the key parameters of the patient. The everyday use of wearables like FitBit, Mi band etc are not only meant for patients, but also for the common everyday use by people. It tracks almost all the parameters required in a particular day. All these are powered by the idea of AI. Currently, there is a newly trending and more advanced form of patient monitoring, i.e. the digital pills, nanorobots, smart fabrics etc which helps in monitoring medication adherence, helps in wound management, and the monitoring of cardiac ailments.^58^ It functions by directly monitoring, which is enabled by brain-computer interfaces and measuring the vital health metrics to keep track of the patient’s emotional, physiological and psychological, and the cognitive states. It is being expected that by 2025, the market for patient monitoring, social health, wearables (which is already trending), telehealth will be adopted by half the population in the developed countries and will be worth more than $350 billion.^59^ The process of digital consultation begins with the patient interacting via the phone using the Wi-Fi or cellular data or the wearable devices enabled with bluetooth and later, transferring the data. This data is then stored in the cloud, computed which then reaches the medical expert, and the review is passed onto the patient through web based-user interface.^60^ In order to reduce the hospital readmissions of the high-risk patients who suffer from illnesses like congestive heart failures, diabetes, pneumonia, hypertension, diabetes and other specific chronic diseases, the CHRISTUS Health Care from Vivify Health took over this pilot project, as such patients often show severe complications post-surgery which results in readmission to the hospital. The patients were provided with a kit that contained a tablet, weight scale, a blood pressure cuff and a pulse oximeter. The results were quite positive, as the patients were able to engage in real time interaction video call with the caregivers, they could answer questions, send their biometric data and watch educational videos. It was observed that, apart from achieving their main goal of reducing the hospital readmissions, they were able to monitor the changes happening in the patient’s body, along with the alert information in case of emergency. It even impacted positively on the revenue of the hospital as well. The average cost to take care of 44 patients (initially it was tested on 44 patients) was $12 937. After the adoption of RPMS (Remote Patient Monitoring System), it dropped down to $1231.^61^ Thus, a 90% decrease in the cost of patient care was observed. Within a pilot duration of one year, it recorded a 95% patient adoption and overall patient satisfaction.^62^ Sensing the success of their project, CHRISTUS Health expanded their RPMS as their initiative to obtain patient satisfaction and adoption. They have also started stepping into EMR and data integration.^63^

### AI in nanotechnology research

The use of AI tools has been expanded to explore its benefits in nanotechnology. AI can be effectively converged with nanotechnology to study the macroscopic and microscopic events occurring in nanosystems. AI could play a key role in studying the behavior of nanosystems and obtain accurate interpretation of the scientific results, thus paving the way for the rational development of nanosystems.^64^ AI has been used in the simulations of nanoscale systems at an atomistic level. It can simulate the way a nanoparticle behaves and aid in the efficient selection of drug carriers, and cut down the cost and labor involved in the development of nanoparticles. Scientists at MIT have explored the use of AI in designing nanoparticles and improving their light scattering properties.^65^ As the field of nanomedicine has evolved over the years and continues to do so, several approaches have been adopted to deliver multiple therapeutic agents in fixed doses. A major challenge with this is that the effect of these drugs in unison depends on the time, dose and is specific to patients. AI could be effectively interfaced with nanomedicine in optimizing the dose to achieve an effective outcome specifically in combination therapy.^66^

### Prediction of an epidemic outbreak

**Figure 6:**
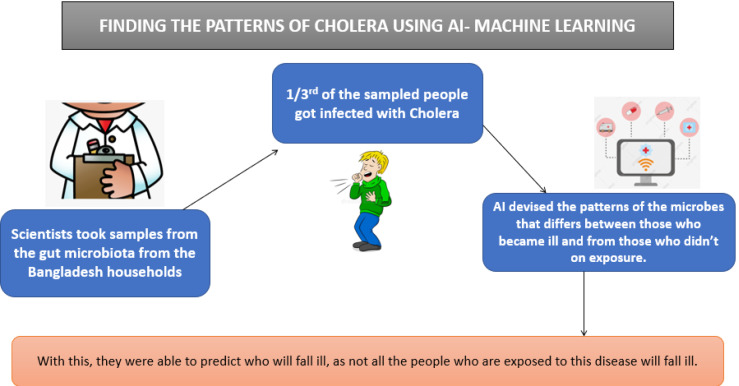
A case of cholera outbreak prediction in Bangladesh using AI.

From predicting weather patterns, helping the doctors diagnose the diseases, changes in the stock market, AI has totally taken over everything in our lives. One of the most astonishing works of AI isthe prediction of an epidemic outbreak.Though, it has not been able to show precise results and is not able to fully bring an outbreak under control, to some extent it has been able to help in a manner to at least take action in the source of time. Using the concept of machine learning, one of the forms of AI, in which the structured data points are used like the location of the epidemic, the count of the reported cases in a particular time, extent to which it is expanding etc, AI has the ability to decide the extent of an outbreak.^67^ It also uses the data provided by the social networking sites like Facebook, Instagram, Twitter etc, where they extract the data through the conversation groups or any forums where the users discuss about the symptoms and cases in their areas or localities. The prediction is done using an algorithm which is created by compiling all the data obtained from gathering the news (from all the languages), the data of the airline ticketing, and also the reports from tracking of the diseases of the plants and animals. The algorithm thus created helps in predicting or at the most simulate the speed of the disease spread to the private and government sectors.^68^ Using machine learning, patterns of cholera were found during its outbreak in Bangladesh, which has been illustrated in Figure 6.^69^

With the help of this machine learning algorithm, at the appropriate time, preventive approaches were taken in order to prevent a huge outbreak.^67^ A Canada based start-up named BlueDot, which was founded in 2013 used AI, machine learning and big data for tracking and predicting the outbreak of infectious diseases. Their engine collects data, every 15 minutes throughout the day, of more than 150 diseases and syndromes across the globe.^70^ The main aim of the engine was to contain the spread of contagious diseases. It collects the data provided by WHO and Centre of Disease Control organizations and reports to the private, government, healthcare and business sectors by providing them a brief synopsis of the disease outbreaks and the risks associated with it, discovered by the AI engine. On the night of 30^th^ December, 2019, BlueDot spotted clusters of “unusual pneumonia” cases happening around the wet and dry markets of Wuhan, China. Immediately, BlueDot alerted their government and private sectors. This was later recognized as the novel coronavirus (COVID-19), which has reported to have infected lakhs of people across the globe and has emerged as a global pandemic. The AI engine had already flagged the Chinese articles reporting 27 cases of pneumonia which had a connection with the markets selling live animals and seafood in Wuhan.^71^ Apart from providing an alert, BlueDot had also accurately identified the cities that had connections with Wuhan by analyzing the data of the global airline ticketing to identify the infected cases travel. It had already reported that international destinations like Tokyo, Singapore, Hong Kong, Phuket, Bangkok, Seoul, and Taipei had the highest number of travelers from Wuhan and that these cities would have a widespread number of cases. As anticipated, these were 11 of the top cities on the list to be infected with COVID-19^72,73^). BlueDot had not only used their system for Corona virus, their AI engine had also been used to predict the outbreak of Zika virus spread to Florida in 2016, 6 months before the spread of the disease. It also determined the 2014 Ebola outbreak in West Africa.^74^

The number of pharmaceutical companies utilizing AI has been increasing over the years. Right from clinical trials to marketing analysis, the value of AI has been increasing due to its adaptive nature and the way it improves the data analysis in the industry. Apart from reducing the time in the trials, it can result in saving costs and render affordable therapies. Companies are able to touch their peak of efficiency in data analysis, by implementing AI in their respective domains. Conventionally, the pharma industry used to face major problems in collecting and processing of medical information, data and medical records being unavailable, and the time and money spent for drug discovery, R&D process etc. Thus, applying machine learning has made the process efficient, seamless, error-free and cost-effective. Table 1 presents the usage of AI in different pharmaceutical sectors.

**Table 1:** Data showing the usage of AI in different pharmaceutical sectors

**Name of the industry**	**Purpose of AI usage**	**Impact of company after AI usage**	**References**
GNS Healthcare	Prediction of a patient’s response to possible drug treatments using AI.	Raised $54.3M in funding for obtaining the machine learning software for this purpose.	^8,29,27^
Insilico Medicine	Generation of new molecular structures & finding of the biological origin of a disease.	Insilico received $20 million in funding for this purpose.	^1,75^
Nuritas	To find & unlock naturally occurring bioactive peptides from food sources.	Nuritas received nearly $28 million as funding for this project.	^58,59^
BioSymetrics	It analyses & integrates different types of biomedical and healthcare data with existing business processes using machine learning.	Studied 334 patients for Alzheimer’s disease. Using the combination of data sources, it led to better diagnosis among the sample group, when compared to one type of data.	^60-63,76^
Numerate	It developed a technology that can simulate the ADME (adsorption, distribution, metabolism & excretion) properties in the drug development process.	Funding was not revealed. But it led to the reduction in the development costs & attrition rates and enabled to a quicker lead design.	^10,64,65,77,78^
Senscio Systems	Uses AI to analyse data related to patient’s health status & self-management behaviour to deliver early detection of possible harmful events.	It had a significant impact on the quality of health care & cost with the improvement of self-management & enabling proactive interferences.	^6,66-68,79^

## Public opinions

Many companies have involved themselves in drug discovery and prediction of drug treatment results in patients. Some are focusing on exploring the advantages of new naturally occurring compounds from food sources over the synthetic compound sources. Many companies claim that, their solutions can be consolidated into the client’s system. Nonetheless, none of the companies have been able to provide a comprehensive detail about the exact method of interaction with their products, and also there has been a lack of transparency about their process of integration. Considering this, it can be perceived that, not all the claims of the companies regarding their application of AI can be trusted and has to be accepted, while maintaining a degree of skepticism about its truth.^80^

## Merits and demerits of AI

Every coin has two sides. In a similar way, AI also can be considered a boon and a bane depending on the way one uses it. In this section, we will be discussing both the merits and the demerits of AI.

### AI- A boon

The first and foremost advantage of AI is that the chances of error are almost negligible. Committing mistakes is very much common among humans. However, machines which possess algorithms and which takes decisions considering previous data, seldom commit mistakes. The business organizations are greatly benefitted by this and are using it in all their domains, due to its ability to solve complicated problems using calculations which minimizes the chances of errors. The organizations are using digital assistants for interacting with their users, which not only saves their time, but also the demand of the user’s businesses also gets fulfilled. So, they need not wait. The systems have been programmed to provide the best kind of assistance to the users throughout the year. In pharma industry, AI helps in reducing the time and cost involved in drug discovery, tackling of rare diseases and also in enhancing the treatment efficiency by personalization of medicines.

### AI- A bane

#### High cost

High cost has always remained a hindrance for the usage of AI. Due to its complexity, it requires huge investments in the installations, repair and maintenance. To adapt to the changing environment, it needs to be updated with the latest software programs from time to time. In case of any breakdown, the procurement cost is quite high and the recovery time is also long.^81^

#### Unemployment

Employment rate in particular is one of the factors influencing the GDP of a country, and unemployment leads to a stagnant GDP. In the future, if humans do not polish and learn new skills, there is a high chance of machines replacing humans. This is one of the major demerits, as the human-intensive requirements and capital-intensive technologies have reduced in some of the industries.^82^

#### Lacks creativity

Machine lacks creativity as the system works in the way it has been commanded to. They cannot really match upto the human brain power, even though they aid in developing and designing. Humans are sensitive intellectuals with the capability of creativity within them, which enables them to think out-of-the-box. Coming to the other aspect of creativity from the human side, if the world gets dominated by AI, and if human are totally dependent on this, then the creativity level of the human beings is bound to decrease.^6^

#### AI in the wrong hands

If the system falls into wrong hands, then it can be misused to any extent, such that it can endanger human lives too. Breaching into other’s privacy and extracting their information has become more easier for the hackers as almost every other person uses AI system in some or the other.^83^

#### Proper operating skills

Specialized skills are required to operate AI and use the system judiciously to ensure the benefits of the system.^84^

#### No enhancement with experience

Human can enhance or improve themselves over the time of experience. But, in the case of AI systems, there is no improvement with experience or time. It has been programmed with a command according to which it will function. If no other command is fed, it will perform the same function again and again. In fact, over the years, it can only lead to wear and tear.^85^

## Methods to curb the limitations

### Creating awareness

The proper usage of AI along with the reach to all the masses can be achieved by creating awareness among the people about the importance and the ways it can be beneficial.^13^

### Creating proper firewall to prevent hacking of the system

Hacking and gaining access to the system can prove to be harmful. So, proper firewalls and hi-tech cyber security should be developed to protect the safety of the system.^86^

### Reduce costs

The costs of the system can be reduced by producing the systems according to the needs of the current scenario as well as the consumers. The tech giants, policy makers, and the department experts are to be consulted before designing an AI for a product or facility.^87^

### Alternate back-up system

The functioning of the AI system cannot be expected to remain the same throughout. In case, during any emergencies, the AI malfunctions suddenly, it could lead to devastating results. Hence, an alternate back-up facility should always be made available, to avoid such devastating results.^88^

## Conclusion and future prospects

AI is a platform which has given an opportunity to explore new horizons, improve and widen our thinking. Even though, these demerits are hindering the usage and productivity of AI, realizing the value of AI, steps are being taken to reduce or prevent such problems and use it to its full potential. AI may not be the total solution to the problem of the transmission of infectious diseases, but it is a tool for an early warning system that can give a timely heads-up to the health professionals and can potentially save thousands of lives, if the disruptive power of AI is used in a good way. Even though the advancements in AI are sky-rocketing, in the domain of pharmacy, it is still at its infancy level. Pharmaceutical industry is not completely ready for AI. According to a recent survey, in case the plan of the researchers goes well according to their expectations, then the AI may take over a great boom to the Global Pharmacy market from $ 2 billion to $ 5.5 billion within no time.^89^ With the sky-rocketing advancements in this field and the boundless applications of AI, it is quite evident that the technology will totally undergo a transformation in the manner in which people work, which will help in quicker, more precise information, increasing the efficiency of the operation and in the innovation of the new products and services. Nonetheless, certain challenges such as data integrity, translation of this data into knowledge, ethical consideration and regulatory approvals may cause a hindrance in the clinical translation of AI in pharmaceutical industry.

## Ethical Issues

Not applicable.

## Conflict of Interest

Authors declare no conflict of interest in this study.
